# Draft Genome Sequences of Two Psychrotolerant Strains, *Colwellia polaris* MCCC 1C00015^T^ and *Colwellia chukchiensis* CGMCC 1.9127^T^

**DOI:** 10.1128/genomeA.01575-17

**Published:** 2018-01-25

**Authors:** Cong Zhang, Wenbin Guo, Yuguang Wang, Xinhua Chen

**Affiliations:** a Key Laboratory of Marine Biogenetic Resources, Third Institute of Oceanography, State Oceanic Administration, Xiamen, People’s Republic of China; b College of Animal Sciences, Fujian Agriculture and Forestry University, Fuzhou, People’s Republic of China; c Laboratory for Marine Biology and Biotechnology, Qingdao National Laboratory for Marine Science and Technology, Qingdao, People’s Republic of China

## Abstract

*Colwellia polaris* MCCC 1C00015^T^ and *Colwellia chukchiensis* CGMCC 1.9127^T^ are psychrotolerant bacteria isolated from the Canadian Basin and Chukchi Sea, respectively. Here, we report the draft genome sequences of *C. polaris* MCCC 1C00015^T^ and *C. chukchiensis* CGMCC 1.9127^T^, which will help reveal how they adapt to cold environments.

## GENOME ANNOUNCEMENT

The *Colwellia* genus was proposed by Deming et al. (1). Many members of this genus are psychrophilic or psychrotolerant (2). *Colwellia psychrerythraea*, the type species of the *Colwellia* genus, is psychrophilic, and how it survives in the cold environment was revealed by its genome sequence (2). Recently, we found a novel psychrophilic species of the *Colwellia* genus and reported its genome sequence, providing the genetic basis for its adaptation to the cold environment (3, 4). Some members of the *Colwellia* genus also produced cold-adaptive proteases, and they are considered to be important to nutrient cycling in cold environments (5). Here, we present the genome sequences of *C. polaris* MCCC 1C00015^T^ and *C. chukchiensis* CGMCC 1.9127^T^, two psychrotolerant strains isolated from the Canadian Basin and Chukchi Sea, respectively (6, 7). *C. polaris* MCCC 1C00015^T^ and *C. chukchiensis* CGMCC 1.9127^T^ were obtained from the Marine Culture Collection of China (MCCC) and China General Microbiological Culture Collection Center (CGMCC), respectively.

Genomic DNA of *C. polaris* MCCC 1C00015^T^ and *C. chukchiensis* CGMCC 1.9127^T^ was extracted and purified using a bacterial genomic DNA kit (Tiangen, Beijing, China), according to the manufacturer’s instruction. Sequencing libraries from the genomic DNA were prepared using the Nextera XT DNA library preparation kit. The whole genomes were sequenced using HiSeq sequencing technology at Biomarker Technologies (Beijing, China), generating 270-bp paired-end reads. The reads were assembled into contiguous sequences (contigs) using the SPAdes algorithm (8). Both genomes exceeded 300× coverage. The draft genomes of *C. polaris* MCCC 1C00015^T^ and *C. chukchiensis* CGMCC 1.9127^T^ yielded 29 and 50 contigs, respectively. The open reading frames of the genomes were predicted by Prodigal (9), and the functions were annotated using BLAST searches of nonredundant (nr) protein sequences from the NCBI-nr database (10). tRNA and rRNA screening was performed using tRNAscan-SE and RNAmmer, respectively (11, 12).

The draft genomes of *C. polaris* MCCC 1C00015^T^ and *C. chukchiensis* CGMCC 1.9127^T^ consist of 4,428,251 and 4,035,164 bases, with mean G+C contents of 37.51% and 41.94%, respectively. A total of 3,784 coding sequences (CDSs), 8 rRNAs, and 53 tRNAs were predicted in the genome of *C. polaris* MCCC 1C00015^T^, and 3,568 CDSs, 11 rRNAs, and 51 tRNAs were predicted in the genome of *C. chukchiensis* CGMCC 1.9127^T^.

*C. polaris* MCCC 1C00015^T^ and *C. chukchiensis* CGMCC 1.9127^T^ possessed 6 and 4 cold-shock proteins and 6 and 1 fatty acid desaturases, respectively. They both possessed the predicted genes involved in the uptake of compatible solute, which acts as an osmoprotectant (13). It might help these two strains survive in cold environments. Both strains encoded the potential cold-adaptive proteases. *C. polaris* MCCC 1C00015^T^ also possessed the genes encoding enzymes that participate in the degradation of starch and chitin.

We predict that the genome sequences of *C. polaris* MCCC 1C00015^T^ and *C. chukchiensis* CGMCC 1.9127^T^ will be useful for revealing how they adapt to cold environments and unraveling their roles in nutrient cycling. Moreover, as cold-adaptive enzymes draw considerable attention in industrial application (14), the genomes may help us find cold-adaptive enzymes that have potential applications in industries.

### Accession number(s).

The whole-genome shotgun projects have been deposited in DDBJ/ENA/GenBank for *C. polaris* MCCC 1C00015^T^ (accession number NBOE00000000) and *C. chukchiensis* CGMCC 1.9127^T^ (accession number NBOC00000000). The versions described in this paper are the first versions, NBOE01000000 and NBOC01000000, respectively.
